# Liver is widely eaten by preschool children in the Northern Cape province of South Africa: Implications for routine vitamin A supplementation

**DOI:** 10.1111/mcn.12931

**Published:** 2019-12-17

**Authors:** Martha E. van Stuijvenberg, Serina E. Schoeman, Jana Nel, Maretha le Roux, Muhammad A. Dhansay

**Affiliations:** ^1^ Non‐Communicable Diseases Research Unit South African Medical Research Council Cape Town South Africa; ^2^ Nutritional Intervention Research Unit South African Medical Research Council Cape Town South Africa; ^3^ Division of Human Nutrition Stellenbosch University Cape Town South Africa; ^4^ Integrated Nutrition Programme Northern Cape Department of Health Kimberley South Africa; ^5^ Burden of Disease Research Unit South African Medical Research Council Cape Town South Africa; ^6^ Department of Paediatrics and Child Health Stellenbosch University Cape Town South Africa

**Keywords:** liver intake, vitamin A, preschool children, routine vitamin A supplementation, Northern Cape province, South Africa

## Abstract

Previous research has demonstrated a virtual absence of vitamin A deficiency and adequacy of vitamin A intake through consumption of liver in preschool children of a community in the Northern Cape province of South Africa where sheep farming is common, and liver, an exceptionally rich source of vitamin A, is frequently eaten. Only 60–75 g of liver per month is needed to meet the vitamin A requirement of preschool children. Because this may have implications for routine vitamin A supplementation, and because liver consumption for the rest of the province is unknown, the study aim was to establish the prevalence and frequency of liver intake in a provincial‐wide survey. An unquantified liver‐specific food frequency questionnaire, covering a period of 1 month, complemented by a 1‐year recall, was administered to mothers of 2‐ to 5‐year‐old children (*n* = 2,864) attending primary health care facilities in all five districts and 26 subdistricts. A total of 86% of children were reported to eat liver, which was eaten in all districts by at least 80% of children. The overall median frequency of liver intake was 1.0 [25th, 75th percentiles: 0.5, 3.0] times per month and ranged from 1.0 [0.3, 2.0] to 2.0 [1.0, 4.0] for the various districts. Based on a previously reported portion size of 66 g, these results suggest vitamin A dietary adequacy in all districts and possibly also vitamin A intake exceeding the Tolerable Upper Intake Level in some children. Routine vitamin A supplementation in this province may not be necessary and should be reconsidered.

**Key Messages**

- Liver is a rich source of vitamin A, and only 60–75 g liver per month is needed to meet the vitamin A requirement of athe preschool child.
- Liver was found to be eaten in all five districts of the Northern Cape province of South Africa.
- The results suggest dietary adequacy of vitamin A in all districts, and possibly also vitamin A intakes exceeding the tolerable upper intake level (UL) in some children.
- Routine high‐dose vitamin A supplementation may not be necessary in this province and needs to be reviewed.

## 1 INTRODUCTION

Vitamin A supplementation is known to reduce morbidity and mortality in vitamin A‐deficient children (Beaton et al., 1993; Imdad, Mayo‐Wilson, Herzer, & Bhutta, 2017), and the WHO recommends periodic high‐dose vitamin A supplementation for children younger than 5 years living in areas where vitamin A deficiency is a public health problem (World Health Organization, 2011). A national vitamin A supplementation programme, which comprises a curative and a routine component, has been in operation in South Africa since 2002 (Department of Health, 2004). Routine supplementation, even though implementation may vary, targets 6‐ to 59‐month children attending primary health care facilities every 6 months. South Africa is a diverse country with varied eating habits, and routine vitamin A supplementation may not be appropriate for all areas. In the Northern Cape province, for example, virtual absence of vitamin A deficiency (<5% with serum retinol <0.70 μmol/L) was reported in preschool children from a low socio‐economic community in the Hantam area, a municipal subdistrict of the Namakwa district, where liver is frequently eaten (van Stuijvenberg, Schoeman, Lombard, & Dhansay, 2012). This was despite high levels of stunting and underweight in the area (van Stuijvenberg et al., 2012) and a national prevalence of vitamin A deficiency of 43% (Shisana et al., 2013).

Sheep farming is the main agricultural activity in the region, resulting in liver being readily available and affordable and thus frequently consumed (van Stuijvenberg et al., 2012).

Liver is an exceptionally rich source of preformed vitamin A (South African Food Database System [SAFOODS], 2016) and can significantly contribute to the vitamin A intake of young children. A dietary intake assessment that quantified vitamin A intake from sheep liver in the same community showed that liver alone provided enough vitamin A to meet the vitamin A requirement of the 2‐ to 5‐year‐old children in this community, even if eaten only once per month (Nel et al., 2014). It is worth noting that in 15% of the children, the Tolerable Upper Intake Level (UL) for vitamin A (Institute of Medicine, 2001) was exceeded through the consumption of liver alone (Nel et al., 2014). Although vitamin A is necessary for normal growth and immune function (Beaton et al., 1993; Sommer & West, 1996), vitamin A will accumulate in the liver if consumed in excess of requirements and may be harmful (Penniston & Tanumihardjo, 2006).

The findings on absence of vitamin A deficiency and vitamin A dietary adequacy through the intake of liver are limited to a particular subdistrict in the Northern Cape province. However, it is likely that liver consumption is more widespread because sheep farming occurs in most of the province. Similar results were found in a community in another district of the Northern Cape, ±500 km east of the study area (Faber et al., 2015). Liver consumption patterns for the rest of the province are, however, not known. Should liver be consumed to the same extent in all of the Northern Cape province, routine vitamin A supplementation in this province may not be necessary. A provincial‐wide epidemiological survey, which aimed to establish the prevalence and frequency of liver consumption amongst preschool children in all districts, was thus undertaken.

## 2 METHODS

### 2.1 Study population and design

This is an epidemiological survey of 2‐ to 5‐year‐old children attending primary health care facilities in the Northern Cape province. The Northern Cape province is the largest of South Africa's nine provinces and covers approximately one third (30.5%) of the country's land area. Yet it is the smallest in terms of population size, encompassing only 2.1% of the total population (Statistics South Africa, 2018). It is characterized by arid conditions with sheep farming being the main agricultural activity. There are five districts with a total of 25 subdistricts (26 at the time of the study). We aimed to include a convenience sample of at least 100 children per subdistrict so that each district would be adequately represented. This was also to allow for meaningful results at subdistrict level, should this information be needed at a later stage. The numbers surveyed per district and subdistrict, as well as the municipalities/towns covered, are in Appendix A.

Dieticians and nutritionists working for the Northern Cape Department of Health were trained by members of the research team to collect the data from the child's mother or caregiver by means of a rapid interviewer‐administered questionnaire. Data were collected as part of normal day‐to‐day activities undertaken by dieticians and nutritionists at primary health care facilities, and the questionnaire took less than 10 min to complete. Anthropometric measurements, which are part of routine measurements during clinic visits, were also obtained. The study was approved by the Ethics Committee of the South African Medical Research Council (Amendment to EC06‐12, 10 March 2011), and permission was obtained from the Northern Cape Department of Health. Verbal informed consent was obtained from the mother or guardian of the child before collecting any information. The survey took place between October 2011 and March 2012.

### 2.2 Questionnaire data

An unquantified liver‐specific food frequency questionnaire, based on a quantified liver frequency questionnaire used previously (Nel et al., 2014) and also more recently (van Stuijvenberg et al., 2019) to assess liver intake in the Namakwa district (Hantam subdistrict), was employed in this survey. Information obtained on liver intake—at both household level and by the child—included a 1‐month recall, complemented by a 1‐year recall for those who did not eat liver during the last month. In this way, a liver intake of less than once a month, which could still make a significant contribution to vitamin A intake, was also captured. For example, liver, eaten only once in 2 or 3 months, was captured as 0.5 or 0.33 times per month.

In addition, data were collected on the age at which liver was introduced into the child's diet and the type of liver (i.e., sheep liver, beef liver, and chicken liver) mostly eaten. Background information was obtained on the mother's educational level, the mother's awareness of the vitamin A supplementation programme, vitamin A supplementation history, current and past breastfeeding, and age at which solid foods were introduced.

### 2.3 Anthropometric status

Height was measured to the nearest 0.1 cm using a portable height measure; the same model from the same supplier was used in all 26 subdistricts (SECA 214 Leicester, Invicta Plastics). Weight was measured to the nearest 0.05 kg using an electronic load cell scale; again, the same model (UC‐321 Personal Precision Health Scale, A&D Company) was used in all subdistricts. All measurements were taken following standard procedures (van Stuijvenberg et al., 2019). *Z*‐scores were calculated using the 2006 WHO growth standards (World Health Organization, 2006). Birth date and birth weight were obtained from the Road‐to‐Health Booklet.

### 2.4 Statistical analysis

Data were captured in Excel and analysed using the IBM SPSS statistical software package, Version 25.0. Continuous data that were normally distributed were expressed as mean (standard deviation) and when not normally distributed as median [25th and 75th percentiles]. Frequency of liver intake, presented as the median number of times per month, was given for the province as a whole and at district level. Categorical data were expressed as percentages. The two‐sided Mann–Whitney *U* test was used to compare the difference in liver intake between those whose mothers had less than 12 years of schooling and those whose mothers had 12 years or more of schooling. *P*‐values < .05 were considered statistically significant.

## 3 RESULTS

Data were collected on 2,864 children. Background characteristics of the study population are shown in Table 1. Biological mothers represented 73.3% of the respondents, and the rest were either the caregiver or guardian. Results for the group as a whole did not differ significantly when only the biological mother as respondent was included in the analyses (data not shown). The term “mother” will therefore be used throughout the text. The majority of the children (88%) were between 2 and 5 years (60 months) old. Stunting, underweight, and wasting were prevalent in 28%, 14.5%, and 5.4% of children, respectively. Only 25% of the mothers had 12 or more years of schooling. Almost all children (88%) had been breastfed in the past or were breastfed at the time of the survey; breastfeeding was continued to a median age of 13.7 months.

**Table 1:** Background characteristics of the study population

Characteristics	n	Mean ± *SD* or median [25th,75th percentiles]	Frequency (%)
Characteristics of mother			
Relationship of respondent to child	2,864		
Biological mother		—	2,099 (73.3)
Caregiver or guardian		—	765 (26.7)
Age (years)	2,864	33.8 ± 11.5	—
Educational level (years of schooling)	2,857	8.8 ± 3.3	—
≤3		—	252 (8.8)
4–7		—	511 (17.9)
8–11		—	1396 (48.9)
12		—	625 (21.9)
>12 (post school qualification)		—	73 (2.6)
Characteristics of child			
Age (years)^a^	2,864	3.51 ± 1.05	—
<2		—	91 (3.2)
2–2.9		—	951 (33.2)
3–3.9		—	801 (28.0)
4–4.9		—	773 (26.9)
≥5		—	248 (8.7)
Gender, % female	2,864	—	1,446 (50.5)
Height‐for‐age *z*‐score^b^	2,864	−1.36 ± 1.23	
% Stunted^c^		—	803 (28.0)
Weight‐for‐age *z*‐score	2,864	−0.87 ± 1.15	
% Underweight		—	414 (14.5)
Weight‐for‐height *z*‐score	2,679	−0.12 ± 1.19	
% Wasted^d^		—	145 (5.4)
Birth weight (g)	2,764	2,940 ± 561	
Birth weight < 2,500 g		—	490 (17.7)
Breastfed or previously breastfed	2,863	—	2,511 (87.7)
Duration of breastfeeding (months)^e^	2,829	13.7 [3.0, 24.0]	
Solid food introduction (months)	2,757	6.0 [3.0, 6.0]	

a The age and gender distribution were similar for all districts.

b *Z*‐scores were calculated using the 2006 WHO growth standards (World Health Organization, 2006).

c Height‐for‐age (HAZ), weight‐for‐age (WAZ), and weight‐for‐height (WHZ) *z*‐scores < −2, respectively.

d WHZ calculated only for children ≤60 months.

e This includes children who have never been breastfed (i.e., a duration of 0 month).

**Figure 1:**
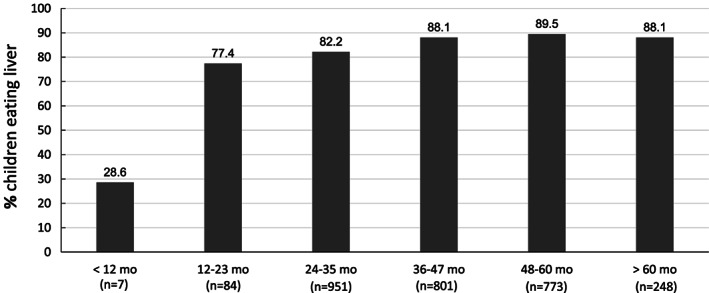
The proportion of preschool children in the Northern Cape province of South Africa who eat liver per age category (*n* = 2,864). The smaller numbers in the age categories <24 and >60 months are because the study focussed on children 24 to 60 months

Liver consumption data for the province and per district are shown in Table 2. In total, 86% of the children ate liver, with 73% eating liver at least once a month and as many as 20% once a week or more. Liver was eaten in all five districts, with the proportion of children eating liver per district ranging between 83% and 93%. The overall median frequency of liver intake was 1.0 [0.5, 3.0] times per month and ranged from 1.0 [0.3, 2.0] to 2.0 [1.0, 4.0] for the various districts. Liver was introduced into the child's diet at a median age of 12 months, and intake increased with the age of the child, plateauing after 36 months (Figure 1). The type of liver most often eaten was sheep liver, either as such or in the form of *harslag* (organ meat); however, chicken and beef liver were also eaten to some extent (data not shown). In all districts, frequency of liver intake (times per month) was higher in households where mothers had <12 years of schooling, compared with households where mothers had ≥12 years of schooling (data not shown). For the John Taolo Gaetsewe district and the Namakwa district, these differences were significant (*p* = .037 and *p* = .020, respectively).

**Table 2:** Liver intake per district at household level and for the child

District	Frequency of liver intake at household level (times/month)	Frequency of liver intake by child (times/month)	Number of children eating liver	Number of children eating liver ≥1×/month	Number of children eating liver ≥1×/week	Age at which liver was introduced into the child's diet (months)^a^
	Median [25th, 75th percentiles]	Median [25th, 75th percentiles]	Frequency (%)	Frequency (%)	Frequency (%)	Median [25th, 75th percentiles]
Siyanda, ZF Mgcawu (*n* = 781)	1.0 [1.0, 2.5]	1.0 [0.5, 2.0]	673 (86.2)	570 (73.0)	123 (15.7)	14.0 [12.0, 24.0]
John Taolo Gaetsewe (*n* = 279)	2.0 [1.0, 4.0]	2.0 [1.0, 4.0]	259 (92.8)	226 (81.0)	83 (29.7)	12.0 [12.0, 24.0]
Namakwa^b^ (*n* = 556)	1.0 [0.5, 2.0]	1.0 [0.3, 2.0]	475 (85.4)	348 (62.6)	73 (13.1)	18.0 [12.0, 24.0]
Pixley ka Seme (*n* = 707)	1.0 [1.0, 2.0]	1.0 [0.5, 2.0]	585 (82.7)	503 (71.1)	126 (17.8)	12.0 [12.0, 24.0]
Francis Baard (*n* = 535)	2.0 [1.0, 4.0]	2.0 [1.0, 4.0]	472 (88.2)	436 (81.5)	164 (30.7)	12.0 [10.0, 24.0]
All districts (*n* = 2,858)	1.0 [1.0, 3.0]	1.0 [0.5, 3.0]	2,464 (86.2)	2,083 (72.9)	569 (19.9)	12.0 [12.0, 24.0]

a Only for children who eat liver; data for this variable available for 2,303 children in total.

The district where previous studies showed lack of vitamin A deficiency and hypervitaminotic liver vitamin A stores in preschool children (van Stuijvenberg et al., 2012; van Stuijvenberg et al., 2019).

The history of vitamin A supplementation, obtained from the child's Road‐to‐Health Booklet, indicated that children received on average 0.9 ± 0.56 supplements per year (this was calculated by dividing the total number of supplements received since birth by the age of the child). Only 16% of children received two supplements per year, the number of supplements recommended by WHO (World Health Organisation, 2011), and 8.4% did not receive any in their lifetime. In response to questions testing the mothers' awareness of the vitamin A supplementation programme, and having been shown a vitamin A capsule, 91.1% of mothers did not know what the capsule contained, 88.7% did not know why it was given to the child, and 88.5% did not know how often the child should receive a capsule.

## 4 DISCUSSION

This study assessed liver intake in preschool children of South Africa's Northern Cape province, a vast and dry region where sheep farming is common. Results show that sheep liver is eaten in all five districts, with an average of almost 90% of the preschool children eating liver.

Liver is an extremely rich source of preformed vitamin A, with lamb's liver containing 7,806 µg RE/100 g, according to the South African Food Composition Database (SAFOODS, 2016). Results of the recently analysed content of sheep livers procured in the Hantam area of Namakwa district showed the vitamin A content to be even higher, at a mean of 11,040 µg RE/100 g, and ranging from 6,308 to as high as 15,200 µg RE/100 g (van Stuijvenberg et al., 2019). The livers of older animals contained more vitamin A than the livers of the younger animals. The exceptionally high liver vitamin A content has important practical implications. The Estimated Average Requirement per day for preschool children ranges between 210 and 275 µg RE (Institute of Medicine, 2001), which means that a child only has to eat 2 to 2.5 g of liver per day (equivalent to a 60–75 g/month) to meet his/her vitamin A needs. This assumes that liver is the only source of vitamin A and does not take into account vitamin A from other sources, such as fortified wheat flour and maize meal. The latter are estimated to contribute an additional 80 µg RE/day to the diet of the preschool children in this province (Faber et al., 2015; Nel et al., 2014).

Liver intake in this survey was used as a proxy for vitamin A intake and hence vitamin A adequacy. The overall median frequency of liver consumption was once a month, ranging between once and twice per month for the different districts. We did not measure portion size in this study, but a previous quantified dietary intake study in the Namakwa district showed an average portion size of 66 g amongst 24‐ to 59‐month‐old children (Nel et al., 2014). Based on the latter portion, the frequency of liver consumption in our study suggests dietary adequacy for vitamin A in all districts in this province. However, it also implies a risk for hypervitaminosis A. A recent study assessing liver vitamin A concentrations by retinol isotope dilution (RID) in 3‐ to 5‐year old children in the Namakwa district, where liver was eaten at a median frequency of only 0.7 times per month, found excessive liver vitamin A concentrations (hypervitaminosis A; ≥1 μmol/g of liver) in 64% of the children (mean 1.13 ± 0.43), which increased to 72% (mean 1.29 ± 0.46) after a high‐dose vitamin A supplement (van Stuijvenberg et al., 2019). The frequency of liver intake observed in the current survey suggests that the hypervitaminosis A seen in the Namakwa district can likely be extrapolated to the other districts in the province.

Too much vitamin A is toxic, and its adverse effects in adults are well documented (Penniston & Tanumihardjo, 2006). However, there is emerging evidence that hypervitaminosis A may interfere with bone formation in young children, even before it reaches toxicity levels. In Zambian children, who had a high prevalence of hypervitaminosis A, bone formation markers improved when dietary preformed vitamin A was reduced (Tanumihardjo, Gannon, Kaliwile, Chileshe, & Binkley, 2019), and in South African preschool children, total liver reserves, as measured by RID, were found to be negatively associated with both height‐for‐age and weight‐for‐age, as well as with bone turnover markers (Tanumihardjo & van Stuijvenberg, unpublished data). Preschool children are at a crucial stage in their development and the potential benefits, and risks of any intervention that could interfere with growth should be carefully weighed before being implemented. It is worth noting that the Northern Cape province has amongst the highest levels of stunting in the country (Labadarios et al., 2000).

Our findings have important implications for the routine vitamin A supplementation programme in South Africa, in that vitamin A supplementation may not be necessary in the Northern Cape province. The literature suggests that high‐dose vitamin A supplementation can be phased out once <5% of the population younger than 5 years has serum retinol values <0.7 μmol/L and when there is proof of dietary adequacy in terms of vitamin A (Palmer, West, Dalmiya, & Schultink, 2012; West, Sommer, Palmer, Schultink, & Habicht, 2015; Wirth et al., 2017). In the Northern Cape province, we demonstrated not only low prevalence of vitamin A deficiency in terms of serum retinol in two separate communities in two separate districts where liver is eaten (Faber et al., 2015; van Stuijvenberg et al., 2012) but, more recently, also hypervitaminosis A in one of the communities (van Stuijvenberg et al., 2019). Furthermore, the present study shows that liver is eaten at a frequency sufficient to ensure adequate intake of vitamin A through the diet in all districts of this province.

The strength of this study is the large number of children surveyed (*n* = 2,864), and that all districts and subdistricts of the Northern Cape province were represented. In nutrition surveys previously undertaken in South Africa, the Northern Cape province was greatly undersampled. Notably, in terms of vitamin A status (serum retinol), the number of children included in the South African National Health and Nutrition Examination Survey (Shisana et al., 2013), the National Food Consumption Survey Fortification Baseline survey (Department of Health, 2007), and the South African Vitamin A Consultative Group survey (Labadarios et al., 1995) for the Northern Cape province was 42, 26, and 497 children, respectively. The 2016 South African Demographic and Health Survey (SADHS) was the only survey to assess liver intake in South African preschool children (National Department of Health, 2019). A total of 578 children countrywide were assessed, of whom 40.5% were reported to have ever eaten liver. Numbers were, however, too small to break down by province, and no data on liver intake for the Northern Cape province were available for comparison. However, the SADHS 2016 did confirm that liver intake in the rest of the country is not as common as the reported 86% for the Northern Cape province in the current study.

Liver vitamin A concentrations, as assessed by RID, is the best indicator, apart from liver biopsy, for measuring vitamin A status (Tanumihardjo et al., 2016). Unlike serum retinol, which cannot detect vitamin A excess, the RID test is able to measure total body stores of vitamin A over the vitamin A status continuum, even in the toxic range. However, because it would be logistically and financially impractical to perform the RID test at the scale of the current study, liver intake was used as a surrogate for vitamin A intake and consequently vitamin A adequacy. Liver intake was assessed by a short liver frequency questionnaire administered by local dieticians as part of their daily activities at the primary health care facilities. This allowed for vitamin A dietary adequacy to be assessed in a rapid, efficient, and inexpensive way and for the whole of the province to be covered. In our recent study, which showed hypervitaminosis A, frequency of liver intake was found to correlate significantly with total liver concentrations in the children (van Stuijvenberg et al., 2019).

The overall lack of awareness amongst mothers in the province of the vitamin A supplementation programme is a concern. Around 90% of mothers were unaware of the content, purpose, and the frequency of distribution of the vitamin A capsules. This may imply that mothers are either not adequately informed about the vitamin A supplementation programme, or that there is a lack of understanding on their part, or both. The vitamin A supplementation coverage rate was also low. Only 16% had received two supplements per year on average, the number recommended by WHO (World Health Organization, 2011).

A limitation of our study is that portion size was not determined. We assumed a portion size of 66 g, which is based on results of a previously published cross‐sectional study in 24‐ to 59‐month‐old children from the Hantam subdistrict of the Namakwa district (Nel et al., 2014), an area typical of the Northern Cape province. However, even if the portion size in the current survey had been smaller, liver would still make a significant contribution to vitamin A intake. It is worth noting that in the recent study where hypervitaminosis A was demonstrated (van Stuijvenberg et al., 2019), the median frequency of liver intake was lower than the median frequency observed in the current survey (0.7 vs. 1.0 times per month).

Another limitation is that the results are representative of the Northern Cape province only and do not pertain to the rest of South Africa, a country very diverse in terms of geography, culture, socio‐economic status, and eating habits. We focussed on the Northern Cape province because of its unique eating habits and because of cumulative evidence of vitamin A adequacy from the Hantam area in the Namaqua district (Nel et al., 2014; Tanumihardjo, Kaliwile, Boy, Dhansay, & van Stuijvenberg, 2018; van Stuijvenberg et al., 2012; van Stuijvenberg et al., 2019). The aim was to see if the latter results could be extrapolated to the rest of the province. In addition, the province has the lowest prevalence of vitamin A deficiency in the country, according to the 1995 national A survey, which is the largest vitamin A survey to date (Labadarios et al., 1995).

Because we were limited in the length of our questionnaire, maternal education was used as a proxy for socio‐economic status. Liver intake in the children was generally higher where the mothers had less than 12 years of schooling, as opposed to where mothers had 12 or more years of schooling. This is similar to what was previously found in the Namakwa district, where liver intake was inversely related to maternal education (van Stuijvenberg et al., 2012), as well as to skilled employment and household income (Nel et al., 2014). Sheep liver is available at both formal and informal outlets in the community and cheaper than meat, which results in the poor buying liver rather than meat. This suggests that the socio‐economically more vulnerable may be less likely to be vitamin A deficient but, at the same time, more likely to develop hypervitaminosis A.

## 5 CONCLUSION

This study showed adequacy of vitamin A through the intake of liver amongst preschool children in all five districts of the Northern Cape province of South Africa. The median frequency of liver intake per month was higher than the frequency in a recent study in the Namakwa district, where hypervitaminosis A was demonstrated. This indicates a risk for hypervitaminosis A in the other districts of this province as well. Against this background and the fact that coverage of vitamin A supplementation in the province is low anyway, we recommend that routine vitamin A supplementation in the Northern Cape province be seriously reconsidered. Resources spent on an unneeded intervention should rather be used to address other nutritional problems, such as the high prevalence of stunting and underweight.

## CONFLICTS OF INTEREST

The authors declare no conflict of interest.

## CONTRIBUTIONS

MES, SES, JN, MR, and MAD conceptualized and designed the study. MES, SES, and JN supervised field procedures. MES analysed and interpreted the data. MES and MAD wrote the paper with input from all authors. The final version was read and approved by all authors.

## APPENDIX A

**Table A1:** The number of preschool children surveyed in each district and subdistrict of the Northern Cape province

District	n	Subdistrict	n	Towns/settlements covered per subdistrict
Siyanda (ZF Mgcawu)	782	Khara Hais	232	Upington, Louisvaleweg, Karos, Raaswater, Kalksloot, Leerkrans
		!Kai Garib	111	Kakamas, Keimoes, Kenhardt, Augrabies, Marchand, Riemvasmaak, Lutzburg
		!Kheis	147	Groblershoop, Topline, Wegdraai, Grootdrink, Boegoeberg/Brandboom
		Mier	92	Askham, Groot Mier, Klein Mier
		Tsantsabane	97	Postmasburg, Postdene, Boichoko, Beeshoek Mine
		Kgatelopele	103	Danielskuil
John Taolo Gaetsewe	279	Gamagara	36^a^	Olifantshoek, Kathu, Dibeng
		Joe Morolong	132	Dithakong, Bothetheletsa, Loopeng, Cassel, Tsineng, Batlharos, Churchill, Vanzylsrus
		Ga‐Segonyana	111	Maruping, Heuningvlei, Bankhara, Mothibastad, Kuruman
Namakwa	557	Hantam^b^	104	Loeriesfontein, Calvinia, Brandvlei, Nieuwoudtville
		Karoo Hoogland	79	Williston, Fraserburg, Sutherland
		Richtersveld	97	Port Nolloth, Alexander Bay, Sandrift, Kuboes, Eksteenfontein, Lekkersing
		Khai‐Ma	47	Aggeneys, Onseepkans, Pofadder, Pella, Witbank
		Kammiesberg	139	Kamieskroon, Garies, Kharkams, Hondeklipbaai, Leliefontein, Kamassies, Kheis, Klipfontein, Nourivier, Paulshoek, Rooifontein, Soebatsfontein, Spoegrivier, Tweerivier
		Nama Khoi	91	Springbok, Bergsig, Steinkopf, Matjieskloof, Carolusberg, Buffelsrivier, Kommagas, Rooiwal, Vioolsdrif, Nababeep, Okiep
Pixley ka Seme	709	Kareeberg/Ubuntu	53	Carnarvon, Victoria Wes, Richmond
		Enthanjeni	130	De Aar, Hanover, Britstown
		Renosterberg	62	Keurtjiekloof, Petrusville, Phillipstown
		Siyancuma	102	Douglas
		Siyathemba	129	Prieska, Marydale, Niekerkshoop
		Thembelihle	146	Hopetown, Strydenberg, Saltlake
		Umsobomvu	87	Colesberg, Norvalspont, Noupoort
Francis Baard	537	Phokwane	191	Jankempdorp, Hartswater, Pampierstad
		Magareng	35	Warrenton
		Dikgatlong	100	Barkley Wes, Delportshoop, Windsorton
		Sol Plaatjie	211	Galeshewe, Florianville, Colville, Phutanang, Ritchie, Vergenoeg, Roodepan, Homevale, Platfontein
Total	2,864		2,864	

a Less than 100 children surveyed in some subdistricts due to smaller population size or lack of staff and infrastructure in these areas.

b The Hantam subdistrict (Namakwa district) where previous studies showed lack of vitamin A deficiency and hypervitaminotic liver vitamin A stores in preschool children (van Stuijvenberg et al., 2012; van Stuijvenberg et al., 2019).

## References

[mcn12931-bib-0001] Beaton, G. , Martorell, R. , Aronson, K. , Edmonston, B. , McCabe, G. , Ross, A. C. , & Harvey, B. (1993). Effectiveness of vitamin A supplementation in the control of young child morbidity and mortality in developing countries. Nutrition policy discussion paper No. 13. Toronto, Canada: Administrative Committee on Coordination‐Subcommittee on Nutrition (ACC/SCN).

[mcn12931-bib-0002] Department of Health (2004). Guidelines for the implementation of vitamin A supplementation. Nutrition Directorate, National Department of Health: Pretoria, South Africa.

[mcn12931-bib-0003] Department of Health (2007). In Labadarios D. (Ed.), National Food Consumption Survey: Fortification baseline South Africa, 2005. Pretoria, South Africa: Department of Health.

[mcn12931-bib-0004] Faber, M. , van Jaarsveld, P. J. , Kunneke, E. , Kruger, H. S. , Schoeman, S. E. , & van Stuijvenberg, M. E. (2015). Vitamin A and anthropometric status of South African preschool children from four areas with known distinct eating patterns. Nutrition, 31, 64–71. 10.1016/j.nut.2014.04.024 25441589

[mcn12931-bib-0005] Imdad, A. , Mayo‐Wilson, E. , Herzer, K. , & Bhutta, Z. A. (2017). Vitamin A supplementation for preventing morbidity and mortality in children from six months to five years of age. The Cochrane Database of Systematic Reviews, 3, CD008524. 28282701 10.1002/14651858.CD008524.pub3 PMC6464706

[mcn12931-bib-0006] Institute of Medicine, Food and Nutrition Board (2001). Dietary reference intakes for vitamin A, vitamin K, arsenic, boron, chromium, copper, iodine, iron, molybdenum, nickel, silicon, vanadium, and zinc (pp. 65–126). Washington DC, USA: National Academy Press.

[mcn12931-bib-0007] Labadarios, D. , Steyn, N. , Maunder, E. , MacIntyre, U. , Swart, R. , Gericke, G. , … Nesamvuni, A. E. (2000). The National Food Consumption Survey (NFCS): Children aged 1‐9 years, South Africa, 1999. Department of Health, Directorate of Nutrition: Pretoria, South Africa. 15806819

[mcn12931-bib-0008] Labadarios, D. , van Middelkoop, A. , Coutsoudis, A. , Eggers, R. R. , Hussey, G. , Ijsselmuiden, C. , & Kotze, J. P. (1995). Children aged 6 to 71 months in South Africa, 1994: Their anthropometric, vitamin A, iron and immunisation coverage status. Johannesburg, South Africa: South African Vitamin A Consultative Group (SAVACG).

[mcn12931-bib-0009] National Department of Health (NDoH); Statistics South Africa (Stats SA); South African Medical Research Council (SAMRC); ICF (2019). South Africa Demographic and Health Survey 2016. Pretoria, South Africa, and Rockville, Maryland, USA: NDoH, Stats SA, SAMRC, and ICF.

[mcn12931-bib-0010] Nel, J. , van Stuijvenberg, M. E. , Schoeman, S. E. , Dhansay, M. A. , Lombard, C. J. , & Du Plessis, L. M. (2014). Liver intake in 24‐59‐month‐old children from an impoverished South African community provides enough vitamin A to meet requirements. Public Health Nutrition, 17, 2798–2805. 10.1017/S1368980013003212 24476795 PMC10282375

[mcn12931-bib-0011] Palmer, A. C. , West, K. P. Jr. , Dalmiya, N. , & Schultink, W. (2012). The use and interpretation of serum retinol distributions in evaluating the public health impact of vitamin A programmes. Public Health Nutrition, 15, 1201–1215. 10.1017/S1368980012000560 22401130

[mcn12931-bib-0012] Penniston, K. L. , & Tanumihardjo, S. A. (2006). The acute and chronic toxic effects of vitamin A. American Journal of Clinical Nutrition, 83, 191–201. 10.1093/ajcn/83.2.191 16469975

[mcn12931-bib-0013] Shisana, O. , Labadarios, D. , Rehle, T. , Simbayi, L. , Zuma, K. , Dhansay, A. , … SANHANES‐1 Team (2013). South African National Health and Nutrition Examination Survey (SANHANES‐1). Cape Town, South Africa: HSRC Press.

[mcn12931-bib-0014] Sommer, A. , & West, K. P. Jr. (1996). Vitamin A deficiency: Health, survival and vision. New York, USA: Oxford University Press.

[mcn12931-bib-0015] South African Food Database System [SAFOODS] (2016). Food composition database, version 2016. Cape Town, South Africa: South African Medical Research Council.

[mcn12931-bib-0016] Statistics South Africa (2018). Mid‐year population estimates 2018. Pretoria, South Africa: STATS SA Available online: www.statssa.gov.za (accessed on 11 June 2019).

[mcn12931-bib-0017] Tanumihardjo, S. A. , Gannon, B. M. , Kaliwile, C. , Chileshe, J. , & Binkley, N. C. (2019). Restricting vitamin A intake increases bone formation in Zambian children with high liver stores of vitamin A. Archives of Osteoporosis, 14, 1–6. 10.1007/s11657-019-0617-y PMC7189610 31254130

[mcn12931-bib-0018] Tanumihardjo, S. A. , Kaliwile, C. , Boy, E. , Dhansay, M. A. , & van Stuijvenberg, M. E. (2018). Overlapping vitamin A interventions in the United States, Guatemala, Zambia, and South Africa: case studies. Annals of the New York Academy of Sciences, 1446, 102–116. 10.1111/nyas.13965 30265402 PMC7999526

[mcn12931-bib-0019] Tanumihardjo, S. A. , Russell, R. M. , Stephensen, C. B. , Gannon, B. M. , Craft, N. E. , Haskell, M. J. , … Raiten, D. J. (2016). Biomarkers of Nutrition for Development (BOND)‐Vitamin A review. Journal of Nutrition, 146, 1816S–1848S. 10.3945/jn.115.229708 27511929 PMC4997277

[mcn12931-bib-0020] van Stuijvenberg, M. E. , Dhansay, M. A. , Nel, J. , Suri, D. , Grahn, M. , Davis, C. R. , & Tanumihardjo, S. A. (2019). South African preschool children habitually consuming sheep liver and exposed to vitamin A supplementation and fortification have hypervitaminotic A liver stores: A cohort study. The American Journal of Clinical Nutrition, 110, 91–101. 10.1093/ajcn/nqy382 31089689

[mcn12931-bib-0021] van Stuijvenberg, M. E. , Schoeman, S. E. , Lombard, C. J. , & Dhansay, M. A. (2012). Serum retinol in 1–6‐year‐old children from a low socio‐economic South African community with a high intake of liver: implications for blanket vitamin A supplementation. Public Health Nutrition, 15, 716–724. 10.1017/S1368980011002126 21859509

[mcn12931-bib-0022] West, K. P. Jr. , Sommer, A. , Palmer, A. , Schultink, W. , & Habicht, J.‐P. (2015). Commentary: Vitamin A policies need rethinking. International Journal of Epidemiology, 44, 292–294. 10.1093/ije/dyu275 25617646

[mcn12931-bib-0023] Wirth, J. P. , Petry, N. , Tanumihardjo, S. A. , Rogers, L. , McLean, E. , Greig, A. , … Rohner, F. (2017). Vitamin A supplementation programs and country‐level evidence of vitamin A deficiency. Nutrients, 9, E190. 28245571 10.3390/nu9030190 PMC5372853

[mcn12931-bib-0024] World Health Organization (2006). Child growth standards. Length/height‐for‐age, weight‐for age, weight‐for‐length, weight‐for‐height and body mass index for age. Methods and development. Geneva, Switzerland: WHO.

[mcn12931-bib-0025] World Health Organization (2011). Guideline: Vitamin A supplementation in infants and children 6‐59 months of age. Geneva, Switzerland: WHO. 24575452

